# Temporal Trends in Outbreaks of Chandipura Viral Infection in India: A Systematic Review

**DOI:** 10.7759/cureus.68097

**Published:** 2024-08-29

**Authors:** Bhavesh Kanabar, Shahenaz Malek, Kiran Piparva

**Affiliations:** 1 Preventive and Social Medicine, Pandit Dindayal Upadhyay Medical College, Rajkot, IND; 2 Pharmacology, Government Medical College, Surat, Surat, IND; 3 Pharmacology, All India Institute of Medical Sciences, Rajkot, Rajkot, IND

**Keywords:** infectious disease outbreaks, encephalitis, chandipura virus, chpv, aes

## Abstract

Chandipura virus (CHPV) has emerged as a significant cause of acute encephalitis in India, especially affecting pediatric populations. This study aimed to analyze the temporal trends, clinical characteristics, and epidemiological features of CHPV infections reported in outbreaks across India. A comprehensive literature search on CHPV cases was conducted using Google Scholar, PubMed, and the Cochrane Library. Original research studies on laboratory-confirmed clinical cases of CHPV infections, available as full-text articles, were included. Data on outbreak characteristics, clinical presentations, diagnostics, and epidemiological factors were extracted and analyzed. Six studies met the inclusion criteria. The review revealed a geographical expansion of CHPV outbreaks across India over time, with a consistent seasonal pattern (May to September) coinciding with the monsoon season. CHPV predominantly affects children under 15 years of age, presenting with rapid-onset encephalitis characterized by high fever, altered consciousness, and seizures. Case fatality rates were alarmingly high, ranging from 28.6% to 78.3% within the first 48 hours of symptom onset. Diagnostic approaches evolved over the study period, with increasing use of molecular techniques. Entomological investigations consistently identified sandflies, particularly *Phlebotomus argentipes,* as potential vectors, though their precise role in transmission remains to be fully elucidated. CHPV is an emerging public health threat, especially for children under 15 years. Early diagnosis is crucial, as CHPV is associated with high mortality within the first 24-48 hours of symptom onset. Challenges include limited long-term follow-up data, potential underreporting of mild cases, and gaps in understanding transmission dynamics.

## Introduction and background

Chandipura virus (CHPV), a newly identified arbovirus, was first isolated by Bhatt and Rodrigues at the Virus Research Centre (VRC) in Pune, India, in 1965. The virus was discovered accidentally while investigating patients with fever in Chandipura village, near Nagpur district in northern Maharashtra, during a dengue or chikungunya virus epidemic [1]. The virus was named "Chandipura" after the location of its discovery [2]. The families of arboviruses include Togaviridae (Alphaviruses), Flaviviridae, Bunyaviridae, and Reoviridae. CHPV belongs to the family Rhabdoviridae, within the order Mononegavirales and the genus Vesiculovirus. These viruses are primarily transmitted by arthropods such as mosquitoes, ticks, and sandflies, although other insects can also serve as vectors. Sandflies of the genera Phlebotomus and Sergentomyia have been proven to be vectors in CHPV transmission [3,4]. CHPV has a notable ability to mutate, enhancing its pathogenicity in causing human infections compared to its genetic relative, vesicular stomatitis virus (VSV) [5]. This virus produces marked cytopathic effects in cell lines as well as in animal models [4].

CHPV symptoms typically present with high-grade fever, abdominal pain, vomiting, altered sensorium, impaired neurological functions, generalized convulsions, decerebrate posture, and coma, mimicking encephalitis [3,6]. Encephalitis is characterized by a diffuse or focal inflammatory process in the brain parenchyma, leading to brain dysfunction [7]. Neurodegeneration following CHPV infection is well-documented, although the specific mechanisms of neuronal death are not fully elucidated. The pathology of encephalitis associated with CHPV infection can be categorized into infection-related encephalitis, directly caused by viral entry into the central nervous system (CNS), and autoimmune-mediated encephalitis, which involves pathological immune responses typically targeting myelin and affecting the brain and spinal cord. Evidence of neutralizing antibodies to CHPV has been observed in serological surveys of domestic animals [3,8].

Acute encephalitis syndrome (AES) is a group of clinically similar neurologic manifestations caused by several different viruses, including Japanese encephalitis virus (JEV), West Nile virus (WNV), chikungunya, and herpes simplex virus (HSV); bacteria such as scrub typhus and tuberculosis; parasites like malaria; spirochetes such as leptospira; fungi; and chemicals/toxins. The well-known viral causes of AES in children include JEV, dengue, HSV, CHPV, WNV, among others [7-9]. In India, Japanese encephalitis (JE) has historically been the leading cause of encephalitis epidemics in children and is a significant contributor to viral encephalitis in eastern and southern Asia [10]. However, several encephalitis outbreaks with high mortality rates remain undiagnosed. Despite its discovery in previous outbreaks, CHPV has received limited attention as an etiological agent. To address this issue, efforts have been made to identify the etiological agents of these encephalitis outbreaks [8]. Currently, the rising number of CHPV infections is becoming an increasing public health concern in India, particularly affecting young children. Given the ongoing outbreak in Gujarat as of July 20, 2024 [11], this study focuses on analyzing CHPV cases identified during outbreaks in India based on published literature.

## Review

Study methodology

The Google, PubMed, and Cochrane databases were searched for research articles on human cases of CHPV up to July 20, 2024.

Study Inclusion

The inclusion criteria for published research articles describing clinical cases of CHPV infection include three key characteristics. Firstly, the studies must identify clinical cases of CHPV infection confirmed through laboratory diagnosis, which can include detecting viral RNA, isolating the virus, identifying IgM antibodies against CHPV in a clinical sample, or observing seroconversion in a convalescent sample. This ensures that only cases with definitive CHPV infection are included in the analysis. Secondly, the criteria allow for both original and epidemiological studies, regardless of whether they are prospective or retrospective in nature. This inclusive approach enables a comprehensive review of available evidence, capturing both carefully controlled prospective studies and valuable retrospective analyses that may offer insights into historical cases and long-term trends. Lastly, only full-text articles are considered for inclusion, ensuring that complete information is available for a thorough assessment of the study's methodology, results, and conclusions. These criteria collectively establish a framework for selecting high-quality, relevant research that provides laboratory-confirmed evidence of CHPV infection while maintaining scientific rigor and comprehensiveness in the review process.

Exclusion Criteria

Research articles other than original research, including review articles, meta-analyses, annual reports, newspaper articles, and studies not conducted on human subjects, were excluded from the analysis.

Results

A total of six studies met the inclusion criteria and have been included in the analysis under the following headings: time trends of CHPV outbreaks in India, demographic and clinical characteristics of CHPV infection (including case definition, diagnosis, and outcomes such as case fatality rate and recovery), and epidemiological analysis of outbreak regions.

**Figure 1:**
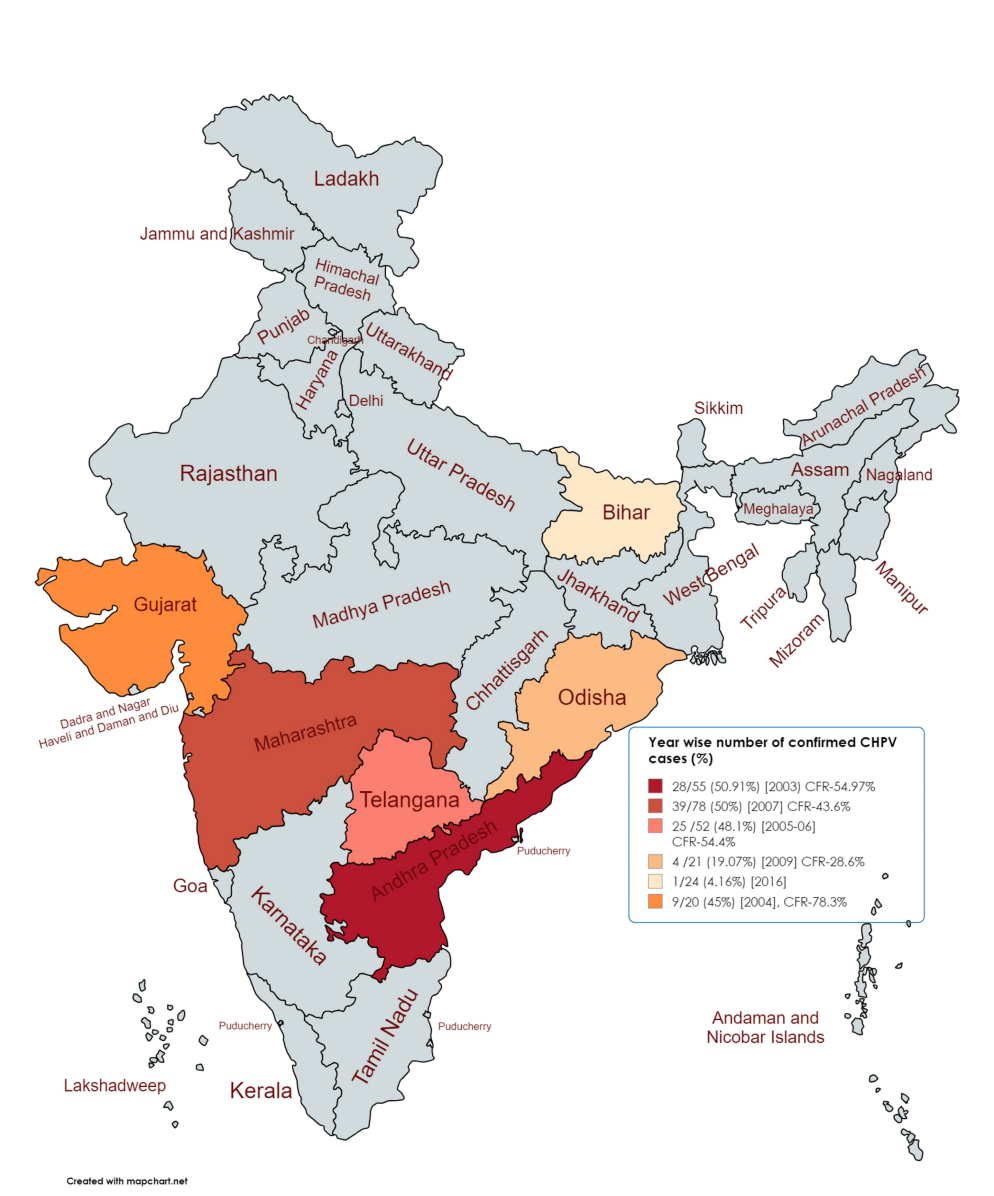
Time trends of the outbreak of Chandipura virus (CHPV) infection in India The figure shows the trends of outbreaks of CHPV infection with Case Fatality Rate (CFR) across various states in India, including Andhra Pradesh (2003), Gujarat (2004), Nagpur (2005 and 2012), North Telangana (2005-2006), Odisha (2009), and Bihar (2018). [8-10, 13-15] Figure source: https://www.mapchart.net/

After the virus was discovered in 1965, it was first isolated in humans from a case of acute encephalitis in 1980 in Madhya Pradesh, India [7]. In 1980, cases clinically diagnosed as viral encephalitis in Raipur, central India, were confirmed to have a CHPV etiology through the isolation of the virus from acute sera [12]. Since then, several outbreaks of CHPV encephalitis have been reported in various states of India, including Andhra Pradesh, Maharashtra, and Gujarat [8]. These studies, arranged chronologically from 2003 to 2018, provide insight into the temporal and spatial progression of CHPV outbreaks across India (Figure 1).

In 2003, CHPV was identified as one of the causes of a large-scale "Epidemic Brain Attack" (EBA) in 10 districts of Andhra Pradesh, with 28 confirmed cases out of 55 suspected cases (50.91%) [10]. In July 2004, a focal outbreak in the eastern districts of Panchmahal and Baroda in Gujarat confirmed CHPV as the cause of encephalitis in 11 out of 20 suspected cases [8]. In 2005, an outbreak occurred in the Bhandara and Nagpur districts, where 7 out of 21 cases were confirmed to be caused by CHPV [13]. Between May 2005 and April 2006, a hospital-based surveillance study of acute encephalitis in children from endemic areas in the North Telangana region of Andhra Pradesh confirmed CHPV infection in 25 out of 52 suspected cases [14]. In 2009, CHPV was confirmed in 21 samples from children suffering from encephalitis in the tribal village of Gudrigaon, Odisha, with 10 of these cases resulting in death [15]. In 2012, an outbreak of AES with high case fatality was reported from several districts in the Vidarbha region of Maharashtra, including Nagpur, Bhandara, Chandrapur, and Wardha. Of the 18 serum samples collected from AES patients, 9 tested positive for CHPV IgM antibody (NIV unpublished data) [7]. In 2018, a single case of CHPV was identified among the AES cases in Gaya district, Bihar [9].

Table 1 summarizes the publications by various authors on CHPV cases reported across different regions in India. Each study had specific objectives, but the main focus was the investigation of AES or stroke outbreaks and the confirmation of CHPV as the etiological agent. CHPV cases were identified from various data sources in the included studies, such as hospital-based surveillance [8, 10, 14] and community-based surveys [9, 15] (Table 1).

**Table 1:** Study characteristics: year, region, study objectives, data source for CHPV cases in India CHPV: Chandipura virus, AES: acute encephalitis syndrome, IgM: immunoglobulin M, N antibodies: nucleocapsid antibodies, CSF: cerebrospinal fluid, AP: Andhra Pradesh.

Authors	Publication year	Region	Study objective	Study design and data source	No. of confirmed CHPV cases
Rao et al. [10]	2004	Andhra Pradesh	To confirm the known causes of "brain attacks" (strokes) in epidemics in 10 districts of AP, and identify CHPV infection	Hospital-based, case-control study among admitted cases at government hospitals)	28 out of 55 cases (50.91%)
Chadha et al. [8]	2005	Eastern districts (Vadodara and Panchamahal) Gujarat	To investigate and confirm the etiology and to describe clinic-epidemiological features of an outbreak of AES among children from Nagpur	Retrospective case series and outbreak investigation (clinical and laboratory data from patients)	9 out of 20 cases (45%)
Gurav et al. [13]	2010	Nagpur, Maharashtra	To investigate an outbreak of AES among children, confirm the etiology, and describe clinic-epidemiological features	Retrospective observational study and outbreak investigation (clinical and laboratory data from hospitalized patients)	39 out of 78 cases (50%)
Tandale et al. [14]	2008	North Telangana, Andhra Pradesh	To elucidate the contribution of CHPV to AES cases in children, seroconversion in recovered cases and to compare the seroprevalences of anti-CHPV IgM and N antibodies in areas of reporting cases	Hospital-based, surveillance study, clinical and laboratory data	25 out of 52 cases (48.1%)
Dwibedi et al. [15]	2015	Gudrigaon, Odisha	To identify CHPV infection causing encephalitis among sudden death of 10 children in a tribal village	Field investigation (door-to-door survey to identify cases) and hospital-based, surveillance study	4 out of 21 tested samples (19.04%)
Singh et al. [9]	2018	Gaya district, Bihar	To investigate an outbreak of acute encephalitis syndrome (AES) and identify the causative agent	Outbreak investigation of patient records from hospital, and field investigation (door-to-door survey) in affected areas laboratory testing of blood and CSF samples	1 case out of 24 cases (4.1%)

A critical aspect of these studies is the confirmation of CHPV cases, which shows considerable variation. The confirmation rates ranged from as high as 50.91% (28 out of 55 cases) in the 2004 Andhra Pradesh study to a single confirmed case out of 24 in the 2018 Bihar study [9,10]. This variation could be attributed to differences in diagnostic capabilities, outbreak severity, or the specific focus of each study.

A summary of the evolution of case definitions and diagnostic criteria, along with the demographics (age and gender) of CHPV cases reported in studies conducted across different regions of India, is presented in Table 2.

**Table 2:** Demographic and clinical characteristics of CHPV cases of included studies CFR: case fatality rate, CNS: central nervous system, JE: Japanese encephalitis, IgM: immunoglobulin M, RNA: ribonucleic acid, ELISA: enzyme-linked immunosorbent assay, JEV: Japanese encephalitis virus, WNV: West Nile virus, HSV: herpes simplex virus, AES: acute encephalitis syndrome.

Author	Case definition	Age	Gender (male: female ratio)	Differential diagnosis and test for exclusion	Outcome and case fatality rate (CFR) %
Rao et al. [10]	Onset of coma and other symptoms such as headache, fever, abdominal pain, diarrhea, seizures, weakness, or loss of consciousness in less than 12 hours. The peak was determined by the Glasgow Coma Scale score or death	5 months to 15 years, with maximal involvement at the age of 2–9 years	1.15:1	Clinical/laboratory evidence of JE, West Nile, dengue, Herpes simplex, enteroviruses, paramyxoviruses, coronaviruses, Varicella, Influenza, Chikungunya, rabies, malaria, typhoid, mycoplasma, leptospirosis, Reye’s syndrome, metabolic disorders	CFR: 54.97%; Recovered cases: 85.19% (23/27) cases recovered within 3 months; Complications: late onset refractory epilepsy in 7.41% (2/27); 4/27 (14.81%) continued to have hemiplegia after 8 months
Chadha et al. [8]	Probable case: sudden onset high-grade fever followed by CNS involvement, altered senses, convulsions, or coma; negative for malaria and other common causes; Confirmed case: presence of viral RNA, IgM antibodies, seroconversion, or virus isolation	2 to 16 years (mean age: 6.03 years)	1:1	Tested for JE virus, West Nile virus, dengue virus, paramyxoviruses	CFR:78.3% 18 out of 23 patients died, while 72.2% (13 out of 18 cases) occurred within 24 hours of onset
Gurav et al. [13]	A case was defined as a hospitalized case, age <15yr, with the acute onset of fever and a change in mental status (including symptoms such as confusion, disorientation, coma, or inability to talk) and/or new onset of seizures (excluding simple febrile seizures)	<15 years, with preponderance in the <5 years age group (46.1%)	1:1.2	Tested for Japanese encephalitis virus, malaria, tuberculosis, and other common bacterial causes	CFR: 43.6% (34 out of 78 patients died), while 60.7% (17 of 28 deaths) occurred within 24 hours of hospitalization
Tandale et al. [14]	Patient aged less than 15 years, presenting with acute onset of fever and central nervous system involvement in the form of one or more symptoms such as altered sensorium, unconsciousness, coma, and convulsions; without signs of meningeal involvement	9 months to 13 years, the majority (35/52, 67.3%) in the 0–4 years age group	1:2.	Patient sera were screened using in-house enzyme-linked immunosorbent assays (ELISAs) for the detection of IgM antibodies against Japanese encephalitis virus (JEV), West Nile virus (WNV), malaria, tuberculosis, and other common bacterial causes	CFR: 54.4% (49 deaths out of 90 total cases) while 76% (19 of 25 deaths) occurred within 48 hours of hospitalization
Dwibedi et al. [15]	A person presenting with sudden onset of fever, altered sensorium, motor weakness, convulsion, abdominal pain, headache, vomiting, or head reeling as a single symptom or in any combination	Cases across all age groups, 40% below 10 years	Not specified	Tested for Japanese encephalitis, dengue, chikungunya, and WNVs	CFR: 28.6% (10 deaths out of 35); 70% of deaths in children below 10 years
Singh et al. [9]	Patients of acute encephalitis syndrome (AES), defined as acute onset of fever with a change in mental status (including symptoms such as confusion, disorientation, coma, or inability to talk) and often with new onset of seizures	1–15 years	1:1.2	Tested for JE, Leptospira, chikungunya, HSV, WNV, Scrub typhus	CFR: 55.5% (5 deaths were recorded out of 9 cases)

Demographic characteristics

An interesting observation is that the age distribution of CHPV cases is consistently skewed toward children, with most studies reporting cases in children under 15 years. Chadha et al. and Gurav et al. focused on children aged 2-16 years and under 15 years, respectively [8,13]. The highest incidence is typically observed in the 2-9-year age group, as noted by Rao et al., while some studies, like Tandale et al., noted a preponderance in children under 5 years [10, 14]. The significantly lower frequency of neutralizing antibodies observed among younger children (under five years) compared to the higher frequency found in the general population suggests that a substantial portion of the pediatric population is susceptible to CHPV [8]. This consistent pattern across studies from different regions and time periods indicates a particular vulnerability of young children to CHPV infection. This age-specific vulnerability warrants further investigation into potential immunological or behavioral factors that may predispose children to CHPV infection or severe disease (Table 2).

Gender distribution shows little variation across studies. While Rao et al. reported a higher incidence in boys [10], Tandale et al. showed a higher proportion of female cases [14]. This inconsistency suggests that gender may not be a significant risk factor for CHPV infection. The clinical presentation of cases during the outbreak is consistent across all studies. The case definitions were based on clinically clustered cases over time, which are also consistent across all the included studies (Table 2).

Clinical characteristics

Case Definition

A case definition is crucial in epidemiology and public health for ensuring consistency in diagnosis, effective surveillance, research analysis, resource allocation, and policy-making. For CHPV infection, case definitions across various studies showed minimal variation, with most utilizing criteria such as "acute onset of fever accompanied by central nervous system/neurological symptoms like altered sensorium, seizures, or coma," or the broader category of "Acute Encephalitis Syndrome" (AES), as observed in real-world data during outbreaks. For example, Rao et al. defined cases by the onset of coma and other symptoms within 12 hours, while Singh et al. used the AES definition [9,10]. The review reveals a consistent clinical picture of CHPV infection, characterized by the acute onset of high-grade fever followed by rapid neurological deterioration. A few studies, such as Tandale et al., included specific age criteria, focusing on children under 15 years, while Gaurav et al. included only hospitalized patients in their definitions [14,15]. Later studies adopted more specific definitions, reflecting an improved understanding of the disease (Table 2).

Criteria Used in Case Definition

The case definition for CHPV infection typically includes the acute onset of fever along with central nervous system or neurological symptoms such as confusion, disorientation, inability to speak, altered sensorium, seizures, convulsions, or coma, as well as conditions like AES or brain stroke. This definition applies particularly to children who require hospitalization for any of these symptoms.

In addition to these criteria, cases from the Gujarat outbreak in 2004 also included gastrointestinal symptoms such as vomiting and diarrhea [8]. Early studies relied more on clinical presentations and basic serological tests, while later investigations incorporated advanced molecular techniques for more precise identification of CHPV.

Most studies indicated that all clinical cases required hospitalization and were managed symptomatically, similar to the approach for AES, with a focus on reducing intracranial pressure. Additionally, a bleeding tendency was observed among hospitalized CHPV cases during outbreaks in Andhra Pradesh, Gujarat, and Nagpur [8, 10, 13].

Diagnosis and Confirmation of CHPV Infection

The consistent use of molecular techniques such as RT-PCR and serological methods across studies reflects the standardization of diagnostic approaches over time. Confirmation of CHPV infection relied on a combination of diagnostic tests conducted on clinical samples. These tests included detecting the presence of CHPV RNA, a crucial diagnostic tool; isolating the virus; identifying IgM antibodies against CHPV, which indicates seroconversion in convalescent samples; and ruling out other viruses to exclude differential diagnoses [8,9,13,15]. The studies emphasize the importance of rapid and accurate diagnosis, given the fulminant nature of the disease.

The case definition for CHPV infection was further supported by excluding other differential diagnoses through testing for IgM antibodies against Japanese encephalitis virus (JEV), West Nile virus (WNV), chikungunya, herpes simplex virus (HSV), scrub typhus, as well as dengue virus and paramyxoviruses [8,11,15]. Additionally, tests were performed to rule out other infections, including leptospira, malaria, tuberculosis, and other common bacterial causes [14].

Demonstration of CHPV RNA was observed in 45% of confirmed cases during the eastern Gujarat outbreak and in 56% of cases during the Nagpur outbreak. Testing for IgM antibodies against CHPV was utilized for samples collected from hospitalized patients and through community surveys during field investigations, indicating seroconversion [8,13].

Case Fatality Rate of Reported CHPV Cases

The most striking feature of CHPV outbreaks is the high case fatality ratio, ranging from 28.6% in Dwibedi et al.'s study to 78.3% in Chadha et al.'s study [8,15]. Overall, most studies report case fatality rates exceeding 50% [8,10] (Table 2).

A notable finding was the rapid deterioration of the disease, with a significant proportion of deaths occurring within 24-48 hours of symptom onset, as reported by Chadha et al., Gurav et al., and Tandale et al. [8,13,14]. The rapid progression of CHPV infections and their high case fatality rates underscore the severity of the disease, necessitating prompt diagnosis, early recognition, and timely intervention.

Significantly lower levels of anti-CHPV neutralizing antibodies were observed among children under 15 years compared to those aged 15 years or older during outbreaks in North Telangana, Andhra Pradesh (2008), and Gujarat (2004), suggesting age-related differences in the immune response to CHPV infection [8,10]. This disparity indicates that younger children may have a less developed or less effective immune response, possibly due to immune system immaturity or limited prior exposure to the virus. This vulnerability could lead to higher susceptibility to severe disease outcomes in younger age groups during CHPV outbreaks.

Recovery

Recovery patterns and sequelae were reported in a study conducted by Rao et al. In this study, many survivors recovered completely within a few weeks to months, with only a small proportion developing neurological complications such as late-onset epilepsy or persistent hemiplegia [10]. Survivors of the outbreak in Odisha (2009), as reported by Dwibedi et al., did not experience any neurological sequelae after recovering from the illness [15]. Similar observations were made during the CHPV outbreak in the Warangal district of Andhra Pradesh (2005-06), where no neurological sequelae were observed [14]. This information is crucial for understanding the long-term impact of CHPV infections and planning appropriate follow-up care.

Epidemiological analysis of included studies

Table 3 provides a comprehensive epidemiological analysis of the included studies on CHPV outbreaks in India, focusing on geographical considerations, seasonality, and entomological investigations.

**Table 3:** Epidemiological analysis of included studies The table insights into the epidemiology of CHPV outbreaks in India, focusing on geographical considerations, seasonality, and entomological investigations from the included studies. CHPV: Chandipura virus, RNA: ribonucleic acid, RT-PCR: reverse transcription-polymerase chain reaction.

Author	Geographical consideration	Season of outbreak	Entomological investigations	Outcome of entomological investigation
Rao et al. [10]	Andhra Pradesh, between 77°-84° East and 13°-19° North	June 1 to August 12, 2003 (monsoon season)	Not done	Not applicable
Chadha et al. [8]	Hilly regions of Gujarat, bordering Madhya Pradesh Located between 22°-23°N and 73°-75°E altitude: Approx. 200 meters above sea level annual rainfall ranges between 441 mm and 1,569 mm	June 9 to July 14, 2004 (pre-monsoon season)	Adult sand flies were collected from houses and peridomestic habitats in the villages with encephalitis cases	81 sand flies in 17 pools tested negative for CHPV RNA by RT-PCR
Gurav et al. [13]	Nagpur, Maharashtra state located between 78°-81°N and 18.5°-21.5°E	June-September 2007 (early monsoon season)	Sandflies collected from houses and around houses of cases	CHPV RNA detected in 1 out of 20 pools of sandflies collected
Tandale et al. [14]	North Telangana region of Andhra Pradesh state Population density 252 per sq km, 81% rural population	May 2005 to April 2006 (late summer and early monsoon)	Not mentioned	Not applicable
Dwibedi et al. [15]	Eastern Ghats, 4000 feet (1212 m) above sea level, at an altitude of 19.9° N and longitude of 84.1° E. The average annual rainfall in the area is 1597 mm The atmospheric temperature varies from 0 °C (December) to 35 °C (May).	Second half of September 2009	Vector survey conducted in domestic and surrounding areas sand flies collected and tested	The presence of *Phlebotomus argentipes *and *Sergentomiya *sp. sand flies found sand flies tested negative for CHPV RNA by RT-PCR
Singh et al. [9]	Located between 24.7213°N and 84.8568°E Average temperature 27-39°C, relative humidity 61%, precipitation 2 mm in June 2016	June 2016	Sand flies collected using light traps and aspirators from affected villages	*Phlebotomus argentipes* sand flies found with a resting density of 5 per man hour sand flies tested negative for CHPV by RT-PCR

Geographical Considerations

CHPV outbreaks have been reported across various states in India, including Andhra Pradesh, Gujarat, Maharashtra, Odisha, and Bihar. In Andhra Pradesh, as noted by Rao et al., cases were reported in an area between 77°-84° East and 13°-19° North. In Gujarat, Chadha et al. reported cases in the eastern hilly region at an altitude of approximately 200 meters above sea level [8,10]. In contrast, in Odisha, Dwibedi et al. observed cases at a much higher altitude of 4,000 feet (1,212 meters) in the Eastern Ghats [15]. This diverse geographical spread indicates that CHPV can affect populations across various topographical settings, from coastal regions to hilly areas.

Seasonality

A consistent pattern emerges regarding the seasonality of CHPV outbreaks. Most studies report outbreaks occurring during the late summer and early monsoon seasons. For instance, Rao et al. and Chadha et al. reported outbreaks from June to August, coinciding with the monsoon season following a hot summer [8,10]. Similarly, Gurav et al.'s study in Maharashtra noted cases from June to September [13]. This seasonal pattern suggests a potential correlation between CHPV outbreaks and climatic conditions, possibly related to vector activity or human behavior during these periods.

Entomological Investigations

Sandflies are implicated as the vector of CHPV due to their presence in outbreak areas and repeated virus isolations. CHPV has been isolated from Phlebotomine sandflies in India and Africa during arbovirus investigations [16]. Entomological investigations were conducted in four out of the six studies, reflecting an increasing awareness of the potential role of insect vectors in CHPV transmission. Chadha et al., Gurav et al., Dwibedi et al., and Singh et al. all reported collecting sandflies from affected areas for analysis [8,9,13,15]. The methods used included light traps, aspirators, and surveys of domestic and peridomestic habitats.

Outcome of Entomological Investigations

The results of these investigations were mixed. While most studies found the presence of sandflies, particularly *Phlebotomus argentipes* and *Sergentomyia *sp., direct evidence linking these vectors to CHPV transmission was limited. Gurav et al. reported detecting CHPV RNA in 1 out of 20 pools of sandflies collected, providing some evidence for the role of these insects in virus transmission [13]. However, other studies, including those by Chadha et al., Dwibedi et al., and Singh et al., reported negative results when testing sandflies for CHPV RNA [8,9,15].

Phylogenetic Analysis

**Figure 2:**
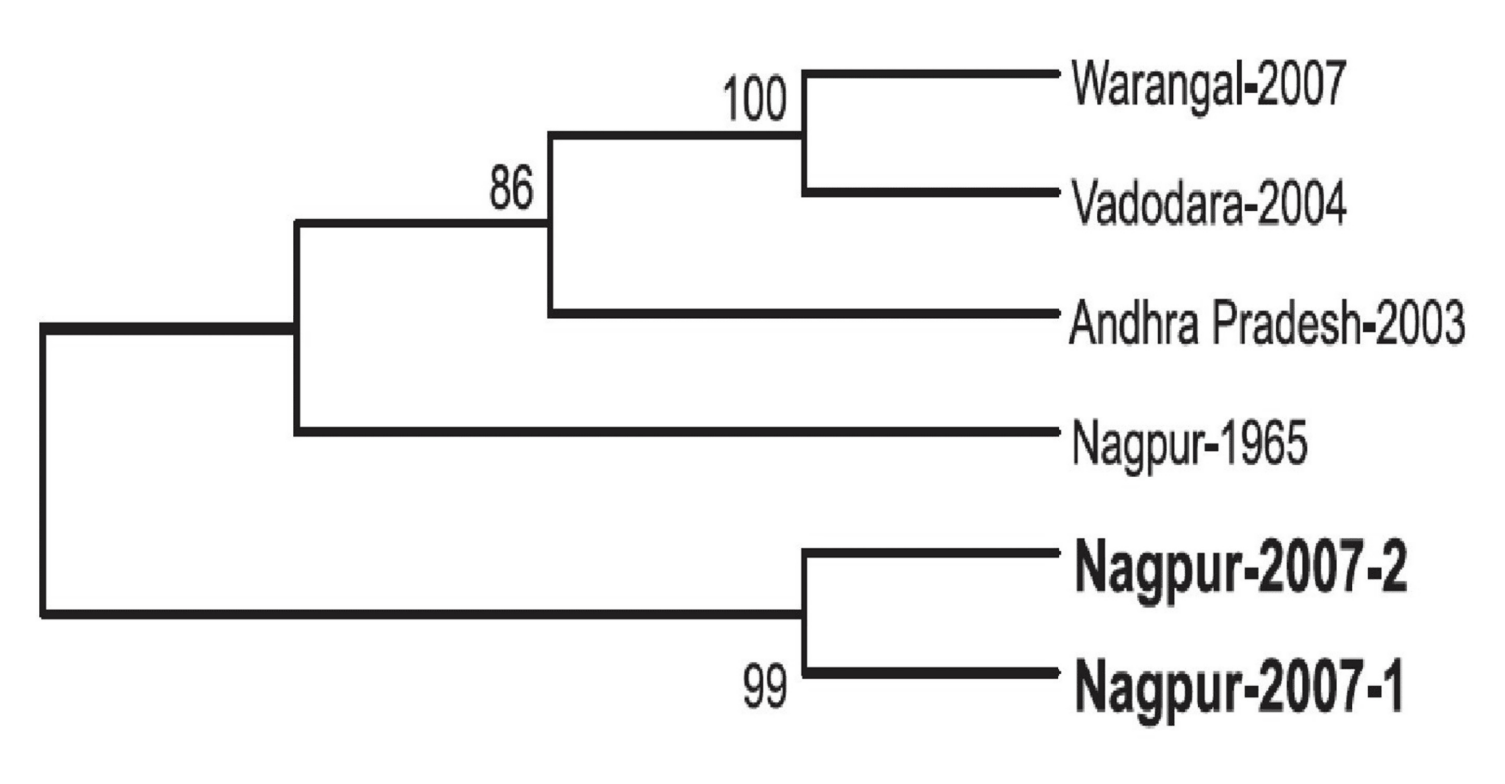
Phylogenetic analysis of CHPV based on partial N gene sequences (2007). Reproduced with permission from reference [13].

Phylogenetic analysis helps to understand the evolutionary relationships and spread of the virus during outbreaks. A phylogenetic analysis conducted on clinical samples obtained during an outbreak in Gujarat (2004) focused on a specific gene (G gene) involved in the outbreak. Based on similarities in the G gene sequences, three major clusters were identified: the first cluster was composed of sequences from Varodara, the second cluster consisted of three sequences from Andhra Pradesh and three from Varodara, and the third cluster included two sequences from Andhra Pradesh and the prototype 1965 strain isolated from Maharashtra State. These clusters suggest genetic relatedness among the viral samples from different geographic regions, with some sequences closely related to historical strains from Maharashtra [8]. Phylogenetic analysis has confirmed that CHPV did not undergo significant mutation from 1965 to 2007 in the Nagpur area or other affected regions [13] (Figure 2).

Discussion

The temporal and geographical analysis of CHPV outbreaks indicates a persistent and concerning spread across multiple Indian states over nearly two decades. The earliest documented outbreak in this review, reported by Rao et al. in 2004, occurred in Andhra Pradesh [10]. The most recent study, conducted by Singh et al. in 2018, identified cases in Bihar [9]. Since early June 2024, Gujarat has reported 78 cases of AES in children under 15, with 28 fatalities. Of these cases, 75 are from Gujarat, 2 from Rajasthan, and 1 from Madhya Pradesh. Testing of 76 samples at NIV Pune confirmed 9 cases positive for CHPV, all of which are from Gujarat, including 5 deaths [11]. This expansion from southern to northern and western India suggests that CHPV could potentially emerge in new regions, possibly due to changes in vector ecology, increased human mobility, or environmental factors.

Case definitions were based on clinical presentations similar to AES, but the diagnosis of CHPV was confirmed through the detection of CHPV RNA or antibodies in all the studies. The exclusion of differential diagnoses was thorough across all studies, highlighting the challenges in accurately identifying CHPV cases, especially in resource-limited settings.

Case detection rates varied across studies, ranging from 45% to 50.91% of suspected cases [8,10,15]. This variation may reflect differences in diagnostic capabilities, outbreak severity, or the specific focus of each study. The consistent use of molecular techniques like RT-PCR and serological methods such as IgM ELISA across studies indicates a standardization in diagnostic approaches over time, potentially improving the accuracy of case identification.

The pathogenicity of the CHPV virus was studied in animals, showing that viremia and high viral titers in the brain were observed in susceptible animals (15 days old), while non-susceptible animals (60 days old) showed undetectable levels of the virus. It was found that IgG seroconversion is necessary to protect the animals from infection [3]. Similarly, children are particularly vulnerable to CHPV infection, with high mortality rates observed in all outbreaks. According to the study by Chadha et al. during the Gujarat outbreak, although IgM antibodies against CHPV were detected in adults, no cases of encephalitis were observed among them [8].

The studies consistently report outbreaks occurring during the late summer and early monsoon seasons, typically between June and September [8-10,13-15]. This seasonal pattern aligns with the breeding cycles of sandflies, the suspected vectors of CHPV, and highlights the importance of targeted vector control measures during these high-risk periods. Ecological studies on CHPV conducted in the Karimnagar and Warangal districts of Andhra Pradesh revealed that blood meal analyses of sandflies showed differences in host preferences: *Ph. papatasi* fed predominantly on humans, cows, and fowls, while *Ph. argentipes* fed on cows. *Sergentomyia *spp. were found to be negative for any of the blood meals tested [17]. The potential role of environmental factors in CHPV transmission is suggested by the consistent seasonal pattern of outbreaks and the geographical characteristics of affected areas. Factors such as temperature, rainfall, and altitude may influence vector abundance and virus transmission dynamics. Future studies should incorporate detailed environmental analysis to better understand these relationships and inform predictive models for outbreak risk.

Study limitation

Even though the study provides valuable insights into the temporal trends and characteristics of CHPV infections in India, it is important to acknowledge several limitations. Firstly, the review included only six studies that met the inclusion criteria. This relatively small number of studies may not fully capture the entire spectrum of CHPV infections in India. Additionally, there was heterogeneity in the study designs, as the included studies varied in their methodologies, case definitions, and diagnostic criteria. This variability makes direct comparisons challenging and potentially affects the consistency of reported data. Furthermore, most studies provided limited information on socioeconomic factors, human behaviors, or local practices that might influence CHPV transmission and susceptibility. Finally, the lack of standardized reporting across studies, particularly in the clinical, epidemiological, and entomological data, made it difficult to conduct comprehensive comparative analyses.

The way forward

The recent increase in AES cases among children in Gujarat (July 2024), confirmed to be due to CHPV infection with a significant number of fatalities, underscores the urgent need for strategic action. Moving forward, it is imperative to implement measures to effectively manage and mitigate this current outbreak.

Research priorities for CHPV should encompass a wide range of critical areas aimed at improving our understanding and management of this emerging pathogen. A comprehensive approach includes elucidating the complete transmission cycle of CHPV, investigating vector ecology and transmission dynamics, and identifying potential reservoir hosts and the precise role of sandflies in virus maintenance and transmission. Host-pathogen interactions, particularly factors contributing to age-specific vulnerability and disease severity, require in-depth study. Developing rapid point-of-care diagnostic tests is crucial for early detection and intervention.

Efforts should be directed toward evaluating the efficacy of antiviral therapies and supportive care protocols to enhance patient outcomes. Additionally, the impact of climate change on CHPV distribution and outbreak potential needs to be assessed, while socio-ecological studies should focus on implementing and evaluating targeted vector control strategies in high-risk areas. Initiating and supporting vaccine research, including the identification of potential antigenic targets, is vital for long-term prevention. Moreover, developing and validating predictive models for CHPV outbreaks based on environmental, climatic, and socio-demographic factors will aid in proactive outbreak management. This multifaceted research agenda aims to address key knowledge gaps and improve our ability to prevent, detect, and respond to CHPV infections effectively.

By addressing these knowledge gaps and strengthening public health interventions, it may be possible to mitigate the impact of CHPV infections and prevent further geographical spread of this emerging pathogen. These actions can help control the current outbreak, prevent future cases, and protect vulnerable populations from the impacts of CHPV and AES.

## Conclusions

This study highlights CHPV as a significant and emerging threat to public health in India, characterized by its propensity to affect children, rapid disease progression, and high mortality rates. The data presented here are invaluable for clinicians and public health officials in recognizing CHPV infections. The geographical expansion of outbreaks over time underscores the urgent need for enhanced nationwide surveillance, targeted vector control, and improved clinical management protocols. Future research should focus on understanding transmission dynamics, developing rapid diagnostics, and exploring potential therapies. The study also emphasizes the need for continued research into more effective treatments and preventive measures to reduce the significant health impact of this emerging pathogen. Public health strategies must prioritize awareness, especially in vulnerable communities. As CHPV continues to pose a threat, a multidisciplinary approach involving entomologists, virologists, clinicians, and public health officials is crucial for effective control and prevention.
